# Genetic markers in clinical subtypes of juvenile idiopathic arthritis

**DOI:** 10.1186/1479-5876-9-S2-P61

**Published:** 2011-11-23

**Authors:** Pierre Le Blay, Catherine Ludwig, Cécile Rittore, Stéphane Soler, Gabriel Tejedor, Vincent Daïen, Nicolas Molinari, Yves-Marie Pers, Jean-David Cohen, Michel Rodiere, Christian Jorgensen, Isabelle Touitou

**Affiliations:** 1 Dépt. Thérapeutique et Médecine Physique Ostéoarticulaire, Unité Génétique Clinique, CHU Montpellier & INSERM U844, Montpellier, France; 2 Unité d’Immuno-Rhumato Pédiatrique, CHU A. De Villeneuve, Montpellier, France; 3 Unité Médicale des Maladies Auto-Inflammatoires (Centre de Référence), Laboratoire de Génétique, CHU Montpellier, Montpellier, France; 4 Institut Universitaire de Recherche Clinique, CHU Montpellier, France

## Introduction

Juvenile idiopathic arthritis (JIA) is the most common children rheumatic disease. This term includes different clinical subtypes that do not have the same presentation, prognosis and therapeutic response. Therefore some studies review this classification, and proposed other criteria as antinuclear antibodies (ANA), age of beginning or uveitis. Our objective was to look for an association between different clinical subtypes of JIA and some single nucleotide polymorphism (SNP), which have already been identify in JIA and other auto-immune diseases.

## Method

We performed a retrospective, monocentric study. Children were classified according to the International League of Associations for Rheumatology (ILAR) classification. Polyarticular and systemic forms, and patients who required biologics were defined as severe. DNA from patients was extracted from a blood sample, and was placed on a FTA elute card. Four SNP in four genes were analysed (*TGF-B* codon 25 rs1800471, *TRAF-1 C5* rs10818488, *STAT 4* rs7574865, et *PTPN22* rs2476601). A Fisher test was performed to analyze the association between those SNP and the different clinical sub types of JIA.

## Results

101 children with JIA were included. Oligoarticular form was the most frequent (68 patients, mean age 4.87 years old (YO)). Polyarticular (FR + and FR -) and enthesitis related arthritis (ERA) forms were less frequent (mean age 5.16 and 11 YO respectively). 18 children developed an uveitis, especially when AAN are positive (14 associated with oligoarticular form). 38 were treated with TNF antagonist (29.4% of oligoarticular, 42.1% of polyarticular and 100% of systemic forms). Genotype CC of *PTPN22* seems to be more frequent in oligoarticular form, but this association is not significant (p=0.12). However, in ANA-positive patients, this association is significant with oligoarticular form, compared to polyarticular JIA (p<0.05). In oligoarticular forms, genotype AA of *TRAF1C5* seems to be associated with the risk of uveitis, but this association is not significant. We did not find association between those SNP and other clinical subtype, disease severity or age of beginning.

## Conclusion

We could not find a significant association between those genetic markers and the different clinical subtype of JIA, but a trend for an association between oligoarticular form and genotype CC of this SNP of *PTPN22.* We are going to try to confirm this association including more children in this study.

